# Enhanced Transdermal Delivery of Concentrated Capsaicin from Chili Extract-Loaded Lipid Nanoparticles with Reduced Skin Irritation

**DOI:** 10.3390/pharmaceutics12050463

**Published:** 2020-05-19

**Authors:** Phunsuk Anantaworasakul, Wantida Chaiyana, Bozena B. Michniak-Kohn, Wandee Rungseevijitprapa, Chadarat Ampasavate

**Affiliations:** 1 Department of Pharmaceutical Sciences, Faculty of Pharmacy, Chiang Mai University, Chiang Mai 50200, Thailand; phunsuk_a@cmu.ac.th (P.A.); wantida.chaiyana@cmu.ac.th (W.C.); 2 Innovation Center for Holistic Health, Nutraceuticals, and Cosmeceuticals, Faculty of Pharmacy, Chiang Mai University, Chiang Mai 50200, Thailand; 3 Department of Pharmaceutics, Ernest Mario School of Pharmacy, Center for Dermal Research, Rutgers—The State University of New Jersey, Piscataway, NJ 08854, USA; michniak@pharmacy.rutgers.edu; 4 Division of Pharmaceutical Chemistry and Technology, Faculty of Pharmaceutical Sciences, Ubon Ratchathani University, Ubon Ratchathani 34190, Thailand; 5 Center for Research and Development of Natural Products for Health, Chiang Mai University, Chiang Mai 50200, Thailand

**Keywords:** capsaicin, chili extract, SLN, NLC, topical delivery system, nanoparticles, irritation

## Abstract

The aim of this study was to develop lipid-based nanoparticles that entrapped a high concentration of capsaicin (0.25%) from a capsicum oleoresin extract. The solid lipid nanoparticles (SLNs) and nanostructured lipid carriers (NLCs) were strategically fabricated to entrap capsaicin without a hazardous solvent. Optimized nanosize lipid particles with high capsaicin entrapment and loading capacity were achieved from pair-wise comparison of the solid lipid mixtures consisting of fatty esters and fatty alcohols, representing small and large crystal-structure molecules combined with a compatible liquid lipid and surfactants (crystallinity index = 3%). This report was focused on selectively captured capsaicin from oleoresin in amorphous chili extract-loaded NLCs with 85.27% ± 0.12% entrapment efficiency (EE) and 8.53% ± 0.01% loading capacity (LC). The particle size, polydispersity index, and zeta potential of chili extract-loaded NLCs were 148.50 ± 2.94 nm, 0.12 ± 0.03, and −29.58 ± 1.37 mV, respectively. The favorable zero-order kinetics that prolonged capsaicin release and the significantly faster transdermal penetration of the NLC attributed to the reduction in skin irritation of the concentrated capsaicin NLCs, as illustrated by the in vitro EpiDerm^TM^ three-dimensional human skin irritation test and hen’s egg test chorioallantoic membrane assay (HET-CAM).

## 1. Introduction

At the present time, the use of the herbal extract, chili oleoresin, is a prerequisite for alternative pain medicine, because of the effectiveness, aroma, and hot sensation of the oleoresin. Chili pepper or chili is a natural crop that contains capsaicinoids, which have played important roles in folk medicine. Capsaicin (8-methyl-*N*-vanillyl-trans-6-nonenamide) is a major active compound in chili which is approved for topical treatment of various forms of neuropathic pain. Capsaicin possesses many therapeutic uses and can act as an analgesic and an anti-inflammatory treatment for gastrointestinal, cardiovascular, and respiratory diseases [1]. Researchers have demonstrated the effectiveness of chili topical formulations for the treatment of chronic pain in many diseases, such as osteoarthritis, rheumatoid arthritis, diabetic neuropathy, and postherpetic neuralgia, as well as psoriasis [2,3]. These diseases have a considerable impact on patients’ quality of life. Commercially available topical products of chili extract generally contain 0.0125–0.075% by weight of capsaicin for the management of neuropathic and musculoskeletal pain [4]. The application of topical products containing low concentrations of capsaicin is usually performed three or four times daily for a period of at least eight weeks [5]. Without active content control in the chili extracts, the effectiveness and side effects of chili topical products fluctuate highly since clinical applications use low dose capsaicin for musculoskeletal pain, and high dose capsaicin for neuropathic pain. Moreover, molecules in chili extract called capsaicinoids consist of dihydrocapsaicin, nordihydrocapsaicin, homodihydrocapsaicin, and homocapsaicin. These molecules share structural and activity similarities with capsaicin, but are not as effective. Thus, the capsaicin concentration should be controlled in topical formulations since its therapeutic and adverse effects are related in a dose-dependent manner [4,6,7]. Products with highly concentrated capsaicin (≥0.075% capsaicin) with a sustained release effect can provide higher effectiveness for severe chronic pain [7], but application may be associated with a burning sensation, leading to severe skin irritation and poor patient compliance [1,8]. These unwanted side effects are due to the rapid uptake of capsaicin in the epidermis and poor penetration into the dermis layer [9], resulting in capsaicin accumulation on the skin surface and in the epidermis. Consequently, external stimuli and transient receptor potential vanilloid subfamily, member 1 (TRPV1) pain receptors located in the epidermis [1,10] become flooded with capsaicin, resulting in a severe skin irritation cascade [1].

Drug delivery systems are used to minimize the problems of certain pharmaceuticals by controlling drug release, targeting drug delivery, and reducing side effects [11]. Solid lipid nanoparticles (SLNs) and nanostructured lipid carriers (NLCs) are lipid-based nanocarriers that have been developed for topical administration route, including controlled drug release, increased drug penetration into the skin, and reduced side effects of concentrated active compounds [11]. The prescription capsaicin strength (8%) in a patch formulation was proven for severe neuropathic pain treatment such as in diabetic neuropathy and postherpetic neuralgia. However, administration under physician supervision for less than one hour is recommended [12]. High entrapment efficiencies of the NLCs for capsaicin were achieved by emulsion evaporation and solvent diffusion techniques; however, both techniques required organic solvents in the preparation [13,14], whereas homogenization and probe sonication protocols can adopt the green chemistry for nanoparticle-fabrication. In previous studies, purified capsaicin powder (>64% capsaicin) was loaded in lipid nanoparticles, resulting in high efficiency of active capsaicin entrapment [10,11,13,14]. Alternatively, microemulsion and nanoemulsion were the delivery systems, in which chili oleoresin extract was incorporated [15,16].

The aim of this study was the development of capsicum oleoresin extract-loaded SLN and NLC lipid-based nanocarriers containing a high concentration (0.25%) of capsaicin for topical application. Capsaicin from the developed chili extract-loaded nanocarriers with particle size less than 300 nm was expected to penetrate into the dermis layer to a much greater extent than the free chili extract incorporated in marketed topical products [17]. The primary expected outcome was the enhancement of the capsaicin skin permeability, resulting in minimizing the contact time of capsaicin in the epidermis, an irritation site. In this study, lipid core composition and preparation methods were the investigated parameters. The SLN and NLC obtained from the selected lipid mixture were characterized for their in vitro release, skin permeation, and retention profiles. Irritation tests of the chili extract-loaded lipid-based nanoparticle and the gel formulation containing the chili extract-loaded lipid nanocarrier were performed using the hen’s egg test chorioallantoic membrane (HET-CAM) assay and the in vitro skin irritation EpiDerm^TM^ test model, respectively.

## 2. Materials and Methods

### 2.1. Materials

Capsaicin standard and capsicum oleoresin extract (chili extract) were obtained from Bangkok Lab and Cosmetic, Ratchaburi, Thailand. Glyceryl behenate (GBH), or Compritol^®^ 888ATO, was obtained from Gattefossé (Lyon, France). Glyceryl monostearate (GMS), cetyl alcohol (COH), stearyl alcohol (SOH), isopropyl myristate (IPM), and jojoba oil were purchased from Namsiang (Bangkok, Thailand). Tween^®^ 80 and Span^®^ 80 were purchased from NOF Corporation (Tokyo, Japan). Acetonitrile, methanol (HPLC grade), glacial acetic acid, ethanol (AR grade), and deionized water were purchased from RCI Labscan (Bangkok, Thailand). Carbopol 940 was purchased from Universal Preserv-A-Chem (NJ, USA). High-viscosity hydroxypropyl methyl cellulose (HV HPMC, viscosity 2600–5600 cP) and isopropanol were purchased from Sigma-Aldrich (MO, USA). Triethanolamine (TEA) was purchased from Fisher Scientific (PA, USA). The fully differentiated human epidermis (EpiDerm^TM^) specimen, EpiDerm culture medium, and 3-(4,5-dimethylthiazol-2-yl)-2,5-diphenyltetrazolium bromide (MTT) assay kit were purchased from MatTek Corporation (MA, USA). Quantikine^®^ human Interleukin 1 alpha (IL-1 α) ELISA kit was purchased from R&D Systems (MN, USA).

### 2.2. Capsaicin Contents in Chili Extract

Ethanolic extract of chili extract (*Capsicum chinense*) was received from Bangkok Lab and Cosmetic, Ratchaburi, Thailand. The accurate amount of capsaicin contents in chili extract was determined using high performance liquid chromatography (HPLC) analysis.

### 2.3. HPLC Analysis

HPLC analysis was performed using an HP1100 chromatograph system and a UV detector set at 280 nm (Hewlett-Packard, Waldbronn, Germany) with a Hypersil ODS (250 × 4.0 mm i.d., 5 µm, Agilent, CA, USA). The mobile phase was the mixture of acetonitrile and 1% acetic acid at a ratio of 1:1 (*v*/*v*), delivered at a flow rate of 1.0 mL/min. The injection volume was set at 10 µL. Validation for capsaicin analysis was performed in terms of accuracy, precision, and linearity. The quantitative determination of capsaicin was obtained from the calibration curve, which had a good linearity at a range of 0.1–100 µg/mL. All samples were measured in triplicate.

### 2.4. Pre-Formulation for Capsaicin-Loaded Lipid-Based Nanoparticles

#### 2.4.1. Lipid Selection for SLN and NLC

Several solid lipids were selected from their physicochemical properties. Simple screening of the solid lipids was performed by melting each lipid with chili extract at the ratio of 10:1. The miscibility test between chili extract and lipid phase (solid and liquid lipid) was further studied. The mixture of chili extract and lipid phase was heated above the melting point of the solid lipid and allowed to cool to room temperature. The homogeneity of the mixture was visually and microscopically observed. The non-crystal lipid mixture was selected for nanoparticle preparation. 

#### 2.4.2. Preparation of SLN and NLC

The preparation of chili extract-loaded SLN and NLC was produced using two methods, hot high-pressure homogenization and probe sonication. Each method consisted of two steps. Firstly, a pre-emulsion was formed by dissolving a chili extract in a melted lipid mixture and Span^®^ 80 under controlled stirring at 70 °C. Both solid and liquid lipids were used in the NLC preparation, whereas only solid lipid was included in the SLN preparation. An aqueous phase was separately prepared by mixing deionized water with Tween^®^ 80 and heated to 75 °C. Then, the water phase was added to the lipid phase and mixed together using a high-speed homogenizer (Polytron^®^, PT-MR 3000, Kinematica AG, Lucerne, Switzerland) at 5000 rpm for 5 min. In the second step, the pre-emulsion was further homogeneously emulsified by passing it through either a high-pressure homogenizer (Avestin, EmulsiFlex^TM^-C3, Ottawa, ON, Canada) at 500 bar for three cycles or sonicating with a probe sonicator (Vibra cell^TM^, Sonics and Materials, CT, USA) for 10 min to form the SLN and NLC vesicles. A sufficient quantity of accurately weighed chili extract to contain 0.25% (*w*/*w*) as the final capsaicin concentration was added to the lipid phase components before melting. In this study, a solid lipid mixture of one fatty acid ester and one fatty alcohol was combined in a factorial design manner at the ratio of 1:3. Next, the SLN and NLC formulations were optimized using lipid phase to surfactant ratios of 1:4 and 1:2 with lipid percentages of 2.5% and 5% in the formulation. Subsequently, the particle size reduction methods were compared between high-pressure homogenization and probe sonication, respectively. Appropriate ranges of the size, surface charge, uniformity, and percentage of capsaicin entrapment efficiency (%EE) of each nanoparticle batch were the considered parameters for selecting the preparation protocols.

#### 2.4.3. Preparation of Chili Extract-Loaded Lipid Nanoparticle Incorporated in Hydrogel

The suitable chili extract-loaded lipid nanocarrier was prepared and incorporated into hydrogel using a mixture of 0.25% (*w*/*w*) Carbopol 940 and 0.25% (*w*/*w*) HPMC. The final concentration of capsaicin in the gel formulation was 0.075% (*w*/*w*) capsaicin.

### 2.5. Characterizing the Chili Extract-Loaded Nanodelivery System

#### 2.5.1. Particle Size, Size Distribution and Zeta Potential Analysis

Particle size, polydispersity index (PDI), and zeta potential of nanoparticles were measured using the dynamic light scattering (DLS) method, Zetasizer^®^, Version 5.00 (Malvern Instruments, Worcestershire, UK). Samples were diluted with filtered DI water at 1:100 dilution before measurement. All experiments were performed in triplicate and expressed as mean ± standard deviation (SD).

#### 2.5.2. Determining Entrapment Efficiency (EE) and Loading Capacity (LC)

EE and LC were determined using an indirect method. Briefly, chili extract-loaded SLN and NLC colloidal suspension were centrifuged at 15,000 rpm for 40 min at 4 °C (SORVALL^®^ SUPER T21 Refrigerated Centrifuge, Sorvall, CT, USA). An aliquot of the supernatant part was dissolved in methanol at 1:20 dilution and filtered to quantify the amount of the capsaicin in the supernatant using HPLC. The initial concentrations of the capsaicin in this experiment were 0.075% and 0.25% (*w/w*). Percentages of EE and LC were calculated using Equations (1) and (2), respectively.

% EE = [(Initial concentration of capsaicin − Capsaicin concentration in supernatant) × 100]/Initial concentration of capsaicin(1)

% LC = [Weight of capsaicin in lipid nanoparticles × 100]/Weight of lipid nanoparticles(2)

#### 2.5.3. Transmission Electron Microscopy Analysis

The morphology of the delivery systems of the chili extract-loaded drug was determined using transmission electron microscopy (TEM, JEM-2011, JEOL, Tokyo, Japan). A single drop of nanoparticles was placed onto a copper grid and stained with 1% phosphotungstic acid. The copper grid was dried at room temperature before analysis. The TEM investigation was performed at 100 kV.

#### 2.5.4. Differential Scanning Calorimetry (DSC) Analysis

Lipid crystallinity, polymorphism, and thermal behavior studies were undertaken using a DSC 822e apparatus (Mettler Toledo, Schwerzenbach, Switzerland) and 5 to 9 mg bulk of lipids, chili extract, physical mixtures, and freeze-dried formulations. Samples were scanned from 20 to 200 °C at a scanning rate of 10 °C/min. An empty aluminum pan was used as reference under nitrogen atmosphere with a flushing rate of 40 mL/min. The melting point, onset temperatures, and enthalpies (ΔH) were evaluated using STARe Software (Mettler Toledo, Schwerzenbach, Switzerland). The crystallinity index (CI, %) was calculated using the Equation (3) [18,19].

CI (%) = [ΔH_formulation_/(ΔH_physical mixture_ × Concentration_lipid phase_)] × 100 (3)

### 2.6. In Vitro Release Kinetics Study

In vitro release of the chili extract-loaded delivery systems was evaluated using a dialysis method [15]. An aliquot of freshly prepared colloidal suspension of the SLN and NLC containing chili extract (0.25% capsaicin) compared with the chili extract solution dissolved in 10% ethanol (as a control) was transferred (1.0 mL) into a 2.5 × 2.5 cm dialysis bag (molecular weight cut-off 12–14 kDa; CelluSep^®^ T4, TX, USA) and then immersed in a chamber. In order to obtain the sink conditions, phosphate buffer saline (PBS; pH 7.4) and absolute ethanol at a ratio of 1:1 (*v*/*v*) (40 mL) was used as a releasing medium. The experiments were conducted at 32 ± 2 °C with gentle stirring at 200 rpm [14]. Samples (3 mL) were withdrawn from the receiving medium and replaced with the same volume of the fresh medium at 0, 0.5, 1, 2, 3, 4, 6, 12, and 24 h. The capsaicin concentrations were measured using HPLC at 280 nm with an 80 µL injection volume. To evaluate the possible release mechanism, zero-order, first-order, and Higuchi models were used to fit the in vitro capsaicin release pattern. 

### 2.7. In Vitro Skin Permeation Study

#### 2.7.1. Drug Permeation

Skin permeation was measured using stillborn porcine skin via the Franz diffusion technique [20]. Full-thickness skin from the dorsal area of stillborn porcine skin was used in this study. The subcutaneous layer was then carefully trimmed off and rinsed with PBS (pH 7.4). The thickness of the porcine skin was 1.55 ± 0.14 mm. Franz diffusion cells with a diffusion area of 2.00 ± 0.42 cm^2^ were used to study in vitro skin permeation. A receptor chamber (12 mL) was filled with the mixture of PBS (pH 7.4) and absolute ethanol at a volume ratio of 1:1 (*v*/*v*) [10,14]. The Franz cells were controlled in a water jacket at 32 ± 2 °C. A 1.0 mL aliquot containing 0.25% capsaicin from freshly prepared chili extract-loaded nanoparticles compared with chili extract solution dissolved in 10% ethanol (as the control) was transferred to the donor chamber. Samples (0.8 mL) from the receptor fluid were collected and immediately replaced with fresh solution at 0, 1, 2, 3, 4, 6, 12, and 24 h. All samples from the receptor fluid were filtered through 0.45 µm pore size membrane filter and assayed using the HPLC system at 280 nm with 80 µL injection volume. The experiments were carried out in triplicate.

The results of permeation through porcine skin are displayed as the cumulative amount of capsaicin permeated (*Q_n_*) per area (µg/cm^2^) versus time (hour), steady state flux (*J_ss_*; µg/cm^2^ h), and permeability coefficient (*K_p_*; cm/h). The cumulative amount of capsaicin was calculated using Equation (4) [20].

(4) $$Q_{n}=V_{r}C_{r}\left(n\right)+\sum_{x=1}^{x=n}\;V_{s}\left(x-1\right)\;C_{r}\left(x-1\right)$$

where *n* is sample time point; *V_r_* and *V_s_* are the volume in the receiver chamber (mL) and the volume of the sample, respectively, collected at the *n^th^* time point (mL); and *C_r_*(*n*) is the concentration of the drug in the receiver chamber medium at the *n^th^* time point (µg/mL).

The rate of capsaicin permeated through the porcine skin was calculated using the slope of the cumulative amount of capsaicin permeated versus time (h) plot. The lag time is the time intercept of the linear portion of the graph. The steady state flux of capsaicin was evaluated using Equation (5) [20].

Steady state flux (*J_ss_*) = (*dQ/dt*)/*A* (5)

where *Q* is the cumulative amount of capsaicin permeated and *A* is the surface area of porcine skin (2 cm^2^).

The permeability coefficient (*K_p_*) of capsaicin and enhancement ratio (*E_r_*) were calculated following Equations (6) and (7), respectively [16,20].

Permeability coefficient (*K_p_*) = Steady state flux/Donor concentration (6)

Enhancement ratio (*E_r_*) = *J_ss_* of formulation/*J_ss_* of chili extract (7)

#### 2.7.2. Skin Distribution

After a 24 h permeation study, skin was washed with deionized water and allowed to dry. Capsaicin concentrations in the stratum corneum (SC) were determined from repeated tape stripping with 20 pieces of adhesive tape using 3M Scotch Magic^TM^ tape (1 × 1 cm). The capsaicin concentration retained in the skin was calculated. After stripping the skin, the sample was cut into small pieces and analyzed for the capsaicin content. The mixture of PBS (pH 7.4) and absolute ethanol at 1:1 (*v*/*v*) was placed in 5 and 2 mL vials for the tape strips and the cut skin, respectively. Then, all samples were sonicated for 30 min and filtered. The filtrate of tape stripping and skin retention was analyzed using the HPLC system at 280 nm with 20 µL injection volume. For both the in vitro release kinetic and the in vitro skin permeation studies, 0.25% capsaicin in chili extract-loaded SLN and NLC formulations were prepared and evaluated in comparison with the chili extract solution containing 0.25% capsaicin in 10% ethanol in water and standard 0.05% capsaicin solution in ethanol. 

### 2.8. Hen’s Egg Test Chorioallantoic Membrane (HET-CAM) Assay

The irritation potential of chili formulations was evaluated by the HET-CAM assay according to the NICEATM-ICCVAM protocol [21]. This is an in vitro alternative method to the skin irritation test [22]. This experiment was slightly modified from previous methods [23,24]. Fertilized hen eggs were obtained from the Faculty of Agriculture, Chiang Mai University. Seven-day-old fertilized hen eggs were used in this experiment and the eggs were incubated in an automatic rotating machine at 37.5 ± 0.5 °C and 62.5% ± 7.5% relative humidity. The shell above the air cell of each egg was opened using a rotating cutting blade attached to a Marathon-3 champion dental micromotor (Saeyang, Korea). Next, the inner membrane directly in contact with the chorioallantoic membrane (CAM) was moistened with normal saline solution and the membrane was then carefully removed using forceps.

Before the experiments, the method was validated with positive and negative controls, which were 1% (*w*/*v*) sodium lauryl sulphate (SLS) and normal saline solution, respectively. The irritation property of chili extract-loaded drug delivery systems (SLN_C and NLC_C) were compared to chili extract solution, and blank nanoparticles (SLN_B and NLC_B). Briefly, 30 µL of test solutions were applied to the CAM and the irritation effect was continuously observed under a stereomicroscope (Olympus, Japan) within 5 min (300 s). The appearance of signs of irritation on the CAM were monitored in terms of vascular hemorrhage, vascular lysis, and vascular coagulation. The time of the first appearance of each irritation sign was registered in seconds and the irritation score (IS) was then calculated using Equation (8) [24].

IS = [(301-H) × 5]/300 + [(301-L) × 7]/300 + [(301-C) × 9]/300(8)

where H is the time point of the first observation of vascular hemorrhage, L is the time point of the first observation of vascular lysis, and C is the time point of the first observation of vascular coagulation, with the time recorded in seconds.

The irritation scores (IS) were classified as no irritation (IS = 0.0–0.9), mild irritation (IS = 1.0–4.9), moderate irritation (IS = 5.0–8.9), and severe irritation (IS = 9.0–21.0). The blood vessels were observed again after 60 min for long-term irritation. The experiment was performed in duplicate.

### 2.9. In Vitro Skin Irritation Test Using a Three-Dimensional EpiDerm Skin Model

#### 2.9.1. MTT Assay on the 3D EpiDerm^TM^ Skin Model

The in vitro irritation study was conducted according to protocol provided by the manufacturer. The EpiDerm^TM^ reconstructed human epidermal (RHE) plate was equilibrated overnight at 37 °C and 5% CO_2_ in a humidified incubator. Then, the chili extract-loaded selected colloidal dispersion and chili extract solution incorporated in a gel formulation containing 0.075% (*w*/*w*) capsaicin, gel base, blank nanoparticle incorporated in gel base, 5% (*w*/*v*) sodium dodecyl sulfate (SDS), and Dulbecco’s phosphate buffered saline (DPBS) were used as the formulation test sample, positive control and negative control, respectively. All tests were applied directly to the top of the 3D tissue and were exposed for 60 min. Next, the EpiDerm^TM^ RHE was rinsed with DPBS then transferred to a fresh medium and incubated for 42 h. All tests were performed twice. After 24 h post-incubation, the culture medium was collected for cytokine release analysis. The fresh medium was then added into the tested EpiDerm^TM^ donor chamber. At the end of incubation (42 h), the RHE tissues were transferred into a 24-well plate prefilled with 300 µL MTT medium (1 mg/mL) per well and placed in a 37 °C, 5% CO_2_ humidified incubator for 3 h. The RHE tissues were removed from the MTT medium. The formazan salt resulting from the MTT dye and viable cell reaction was extracted from the tissues by transferring them to a 24-well plate containing 2 mL of isopropanol. The submerged tissues were shaken at 120 rpm at room temperature for 2 h. After the extraction period, the optical density (OD) of the extracted formazan was determined by transferring 200 µl of each extraction solution into a new, optically clear 96-well plate. Isopropanol was used as a blank vehicle. The OD values were determined using a spectrophotometer at 570 nm. The percent cell viability was calculated using Equation (9) [25,26].

% Viability = [OD (sample) × 100]/OD (negative control)(9)

#### 2.9.2. ELISA Assay

The release of IL-1α after a 24-h incubation period with the test samples from the EpiDerm RHE tissue to the culture medium was collected and quantified using Quantikine^®^ ELISA kits according to the assay protocol provided by the manufacturer. UV-visible spectrophotometric measurements were performed at 450 nm using a microplate reader (Biotek Power Wave X, VT, USA) [25,26].

### 2.10. Statistical Analysis

Data were collected in three independent experiments and are shown as mean ± SD. Data were analyzed using the *t*-test, ANOVA, and Tukey’s multiple comparison test in SPSS software, version 22.0 (IBM SPSS, Armonk, NY, USA); *p*-values less than 0.05 were considered statistically significant [27].

## 3. Results and Discussion

### 3.1. Capsaicin Contents in Chili Extract

The HPLC chromatograms of capsaicin and dihydrocapsaicin showed the retention times at 7.315 and 9.932 min, respectively (supplementary data). Capsaicin was found as the major compound and the calculated capsaicin content in chili extract was 20.55%.

### 3.2. Pre-Formulation for Capsaicin Loaded Lipid-Based Nanoparticles

#### 3.2.1. Lipid Selection for SLN and NLC

In the selection of appropriate lipid carriers for a lipophilic compound such as capsaicin in chili extract, the lipid core for the entrapment should provide high capsaicin solubilization. Recommended lipid carriers for a lipophilic drug were medium-chain (6–12 carbon atoms) and long-chain (13–21 carbon atoms) fatty acids rather than short-chain fatty acids (fewer than five carbon atoms) [28]. The mixtures of long chain solid lipids were paired between the fatty acid esters, i.e., GBH or GMS, and fatty alcohols, i.e., SOH or COH. Solid lipid types were chosen corresponding to the difference in melting point (m.p.), required hydrophilic lipophilic balance (HLB), and crystallization pattern of the inner core. The combined solid lipids from different groups caused an amorphous form which could decrease lipid crystal inner space. The rearrangement of the inner structure of the lipid crystal or an increase in lipid network heterogeneity was further enhanced by adding a liquid lipid into the network to obtain the NLC carrier. The mixture of different kinds of lipids can create a large empty space to accommodate the drug [29]. The lipid compositions were selected based on their compatibility with a chili extract as observed from the physical and microscopic characteristics of the solidified lipid samples after melting (supplementary data).

#### 3.2.2. Characterizing the Chili Extract-Loaded Nanodelivery System

For SLN fabrication, the solid lipid ester and solid lipid alcohol at a 1:3 ratio were emulsified with the aqueous phase containing a mixture of Tween^®^ 80 and Span^®^ 80 at the corresponding required HLB of approximately 8–10 to produce oil in water-based nanoparticles with sufficient stability and dispersity for topical products [30]. In this study, the ratios of the lipid phase to the surfactant were 1:4 and 1:2, and the resulting increase in lipid mixture proportion from 2.5% to 5.0% led to an increase in inner phase viscosity and a larger particle size, i.e., from 130 to 180 nm, with a reduction in drug loading capacity, as has been reported previously [17]. The non-ionic surfactants used in these formulations (Tween^®^ 80 and Span^®^ 80) were reported to stabilize colloidal systems due to the steric hindrance effect, which prevents phase separation with a zeta potential of −18 to −44 mV [19]. The liquid lipid of NLC formulations was selected from jojoba oil and IPM at a 7:3 ratio of solid lipid to liquid lipid, the suitable ratio from the previous studies [17,31]. Regarding solubility, capsaicin was dissolved in jojoba oil and IPM at the concentrations of 2.80 ± 0.28 and 5.56 ± 0.02 mg/mL, respectively. When the NLC formulations were optimized, particle sizes of 157.66 ± 4.34 and 140.24 ± 3.53 nm, size distributions of 0.22 ± 0.04 and 0.25 ± 0.03, and zeta potentials of −47.24 ± 1.68 and −58.98 ± 2.88 mV were obtained from jojoba oil and IPM, respectively. The higher solubility of capsaicin and smaller NLC particles using IPM as the liquid lipid were the reasons for selecting IPM. The particle sizes of SLN and NLC at the 1:4 ratio of lipid phase to surfactant were below 200 nm, with a narrower size distribution than with the 1:2 ratio. The characteristics of the SLN and NLC formulations upon the optimization of lipid compositions are shown in Table 1. Furthermore, the characteristics of SLN and NLC with a 1:2 ratio of the lipid phase to surfactant were a larger size and broader size distribution than those with a 1:4 ratio (data not shown).

In the first step of SLN and NLC preparation, the pre-emulsion was prepared with the beaker method, resulting in a coarse emulsion with average particle sizes in the micrometer range. The second step involved particle size reduction using hot high-pressure homogenization or probe sonication. As expected, high pressure homogenization reduced the particle size approximately ten-fold for SLN and two-fold for NLC with a narrower size distribution compared to size reduction with probe sonication (Table 2).

#### 3.2.3. Entrapment Efficiency (EE) and Loading Capacity (LC)

Two strengths of the chili extract, i.e., 0.075% and 0.25% capsaicin, were loaded in the lipid nanoparticles. The EE and LC percentages of each formulation are shown in Table 3. The loading effectiveness of an active component from a natural origin such as chili oleoresin into a carrier is a complex system due to various interactions, mainly hydrophobic interactions, between lipid core structures and the chemical compounds in natural plants [32]. The results show that the formulations composed of GMS and COH as the solid lipids yielded higher %EE and %LC, and the NLC formulations also exhibited significantly higher %EE and %LC than SLN-based formulations due to the less-ordered lipid structure and more amorphous area provided in NLC than in SLN. Moreover, the liquid lipid in NLC increased chili extract solubility in the lipid phase, resulting in higher entrapment of the chili extract in a homogeneous distribution in the carrier structure [33]. In addition, loading capacity refers to the percentage of drug incorporated into the lipid nanoparticles, the high values of %LC showing that capsaicin was a rather lipophilic compound that preferably solubilized in a lipid matrix [34]. According to the results in Table 3, greater entrapment and loading capacity highly depend on liquid lipid miscibility. Wang et al. (2017) presented the Box–Behnken design for capsaicin-loaded NLCs preparation experiments from the preselected GMS, Miglyol^®^ 812 and Tween 80 as the main compositions. The high entrapment efficacy of 91.6%, average particle size of 119 nm and −17.0 mV zeta potential were reported for the 0.15% capsaicin in capsaicin-loaded NLCs [10]. From our study, the final core-compositions yielded not only high EE with approximately 140 nm particle size, but also negative charges lower than −30 mV, indicating excellent colloidal stability [35]. 

#### 3.2.4. TEM Investigation

**Figure 1:**
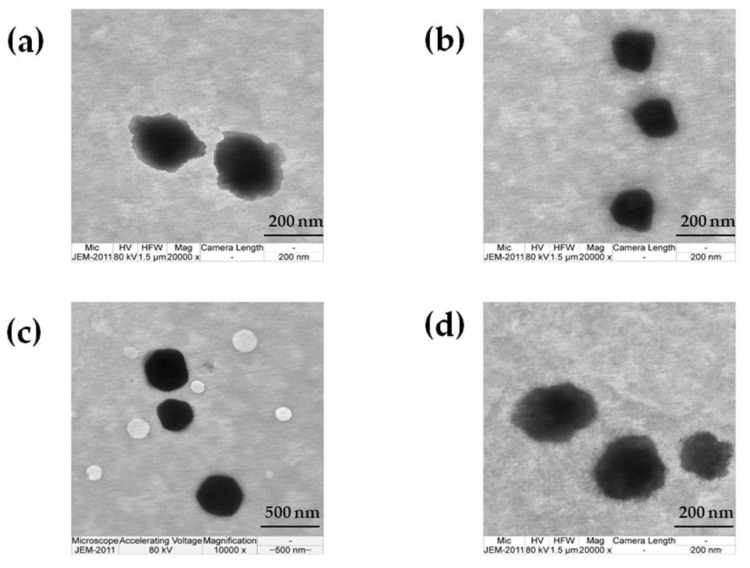
Electron microphotographic images of solid lipid nanoparticles (SLN) and nanostructured lipid carriers (NLC); (**a**) blank SLN, (**b**) chili extract-loaded SLN, (**c**) blank NLC, and (**d**) chili extract-loaded NLC.

The morphology of the blank nanoparticles and chili extract-loaded nanoparticles is shown in Figure 1. The obtained nanoparticles were spherical in shape and homogeneously colored with the particle size distributed approximately from 200 to 300 nm. The morphology of the chili extract-loaded nanoparticles (Figure 1b,d) was similar to the blank nanoparticles (Figure 1a,c).

#### 3.2.5. Differential Scanning Calorimetry (DSC) Analysis

**Figure 2:**
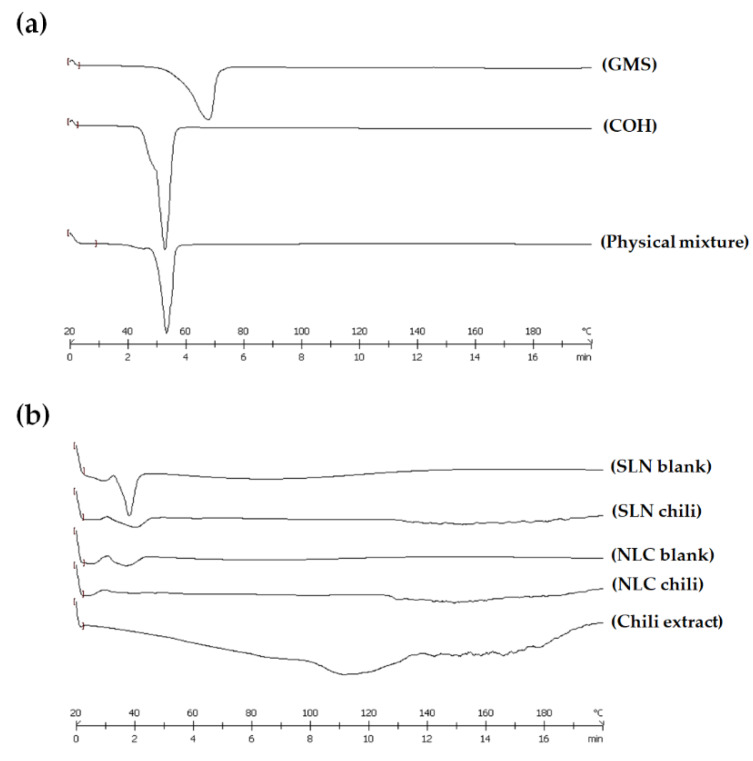
Differential scanning calorimetry (DSC) thermograms of (**a**) the pure solid lipid and physical mixture, and (**b**) freeze-dried formulations. Abbreviations: SLN, solid lipid nanoparticles; NLC, nanostructured lipid carriers; GMS, glyceryl monostearate; COH, cetyl alcohol.

DSC was utilized to investigate the thermal behavior of materials composed of lipid nanoparticles. The DSC thermograms of GMS, COH, physical mixture, and freeze-dried formulations in different ratios of solid lipid and liquid lipid are shown in Figure 2a,b, respectively.

The melting peaks of solid lipids (GMS and COH) revealed the solid character of the lipid matrix at room temperature. The melting peaks of GMS and COH were at 66.77 and 51.35 °C, respectively. A physical mixture was prepared using mixed solid lipids (GMS and COH at a 1:3 ratio), heated to 75 °C and cooled down to room temperature. The melting peak of the physical mixture still presented a sharp melting endotherm of the lipid, with a shift in the temperature from the bulk lipid to 52.15 °C, showing the domination of COH. The DSC thermograms of the pure solid lipid and physical mixture are shown in Figure 2a. The wax:oil (W:O) ratios of the solid lipid and liquid lipid were 10:0 (SLN) and 7:3 (NLC). The crystallinity of the freeze-dried formulations at different ratios of solid lipid and liquid lipid demonstrated that the liquid lipid decreased the solid character of the lipid matrix in the formulation. The depression in crystallinity was influenced by the liquid lipid level, which provided an amorphous form or imperfection to the lipid matrix. Chili extract-loaded SLN and NLC were produced along with a freeze-dried formulation to investigate the thermal behavior. The DSC thermograms of the chili extract and the freeze-dried formulations of chili extract-loaded SLN and NLC are shown in Figure 2b. 

The DSC thermogram of chili extract exhibited a flat peak onset from 100 to 130 °C, which was the endothermic peak of water evaporation. A decline was observed in the melting endotherm for the freeze-dried chili extract-loaded SLN and NLC. The DSC peaks of the chili extract-loaded SLN and NLC were different from the peaks of chili extract and SLN-NLC blank formulation. It was demonstrated that the lipid crystals were disrupted, and the crystalline properties were reduced because of the interaction between the chili extract and the lipid matrix, indicating that the chili extract had dissolved in the lipid matrix. The formulation including not only chili extract but also liquid lipid (NLC) revealed an amorphous form or imperfect lipid phase, as a result of improving the drug’s entrapment efficiency, in agreement with other reports [18,31]. The calculation of the crystallinity index is shown in Table 4.

### 3.3. In Vitro Release Kinetics Study

**Figure 3:**
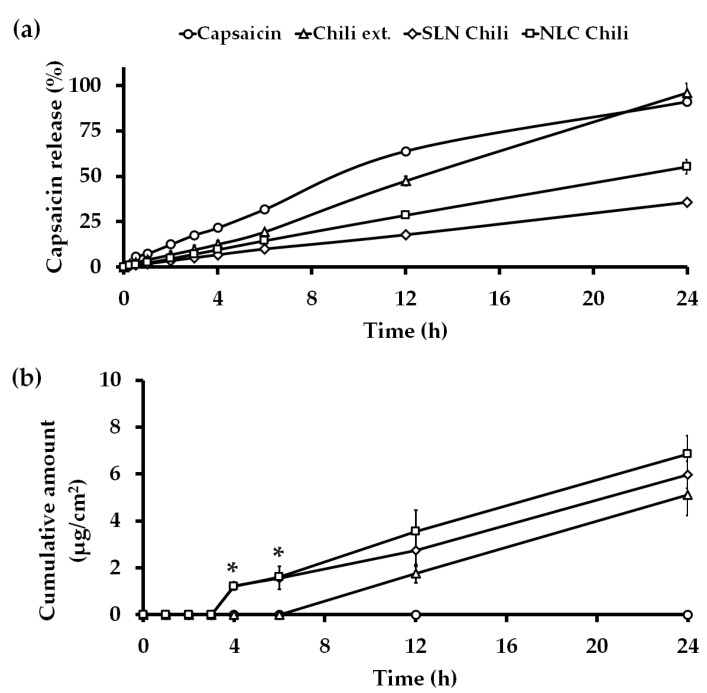
(**a**) In vitro release study and (**b**) in vitro permeation study of capsaicin solution (-○-), chili extract solution (-∆-), SLN chili (-◊-) and NLC chili (-□-) at various times within 24 h. (n = 3). Abbreviations: SLN, solid lipid nanoparticles; NLC, nanostructured lipid carriers. * The value represents a significant difference (*p* < 0.05) from other formulations.

The release profiles from the delivery system of the chili extract-loaded nano-formulation with highly capsaicin concentration (0.25%) are an important set of data since a sustained release drug delivery system can decrease the unwanted burning sensation of capsaicin [36]. In this study, the capsaicin release profile was determined over a period of 24 h using the dialysis method in PBS (pH 7.4):ethanol (1:1) as the medium at 32 ± 2 °C and 200 rpm. The capsaicin release is shown in Figure 3a. The zero-order kinetic model matched for all formulations with the regression values (R^2^) 0.9952, 0.9918, 0.9952, and 0.9941 for capsaicin, chili extract solution, SLN chili and NLC chili, respectively. The mathematical models of the capsaicin release profile from each formulation are shown in the supplementary data. The capsaicin release profile of the chili extract solution was exhibited for nearly 24 h because the original 10% ethanol may have led to capsaicin microprecipitation in the dialysis bag and therefore acted as a reservoir for the active compounds, thus resulting in the zero-order release kinetics, whereas the capsaicin in NLC and SLN dissolved in the lipid core and exhibited greater sustained release kinetic compared to the controls. In addition, the initial 0.25% concentration of capsaicin was prepared to be equivalent to the concentration in the nano-formulation, thus this moderately low concentration reduced the initial burst effect and caused prolonged release of capsaicin solutions [37]. The NLC formulation produced an increase in the amount of capsaicin released when compared with the SLN formulation because of the liquid lipid in NLC [38]. The zero-order kinetic model is related to drug dissolution from several types of pharmaceutical delivery systems, such as matrix tablets, osmotic and transdermal systems [39]. The zero-order kinetic model is the ultimate goal of all controlled release drug delivery systems, since drug efficacy can be maximized while dose frequency and toxicity can be minimized [36].

### 3.4. In Vitro Skin Permeation Study

The permeability of the chili extract-loaded nanodelivery system through stillborn porcine skin was determined using the Franz diffusion technique. The capsaicin concentrations in the stratum corneum and those retained in the dermal layer were evaluated using tape stripping and by conducting a retention study, respectively. The cumulative capsaicin permeation through stillborn porcine skin was assessed over 24 h and is presented in Figure 3b and Table 5. The steady state flux or permeation coefficient of capsaicin in this study were different from the previous reports [10,14,16]. The permeation parameters were dependent on the animal skin model and the thickness of the skin [40]. The capsaicin from the chili extract-loaded lipid-based nanocarrier permeated the skin to a greater extent than the chili extract and capsaicin solution. The skin permeation flux of capsaicin from chili extract loaded in 2.5% lipid of NLC (0.45 ± 0.16 µg/cm^2^ h) and SLN (0.37 ± 0.12 µg/cm^2^ h) were significantly (*p* < 0.05) higher than the chili extract solution as a control (0.00 ± 0.00 µg/cm^2^ h) until 6 h. However, the enhancement ratio of capsaicin after 24 h from the lipid nanoparticle dispersion was not statistically significant (*p* > 0.05) compared to the control. Even though the in vitro release study of the nanodelivery systems revealed sustained release, the permeation profiles of the chili extract-loaded lipid nanocarriers were contrasting, since capsaicin from the nanocarriers permeated through the skin faster and to a greater extent than the chili extract solution with the lag time, and the permeability coefficient of chili extract-loaded nanoparticles was significantly (*p* < 0.05) different compared to the chili extract solution due to the characteristics of lipid nanocarriers, such as lipophilicity and a particle size below 200 nm. Nanocarriers can form a monolayer on the skin surface, causing an occlusive effect on the skin and enhancing the permeation of the compounds by loosening the corneocyte packing and the opening inter-corneocyte gaps. In addition, the penetration of NLCs was affected from the complex interactions of the lipids and surfactant of the NLC with the lipids in the skin layers [10,35]. As shown in Figure 3b, chili extract and capsaicin solution exhibited poor penetration in the initial time until 6 h, resulting in higher capsaicin accumulation in the stratum corneum; this could cause irritation as a side effect. A high capsaicin concentration in viable epithelial skin has been reported to cause severe skin irritation [14]. There is evidence that the TRPV1 receptors at the nerve endings in the epidermis regulate the irritation responses. When free capsaicin, a stimulus, binds to these receptors, an irritation cascade occurs in the skin [1,41].

**Figure 4:**
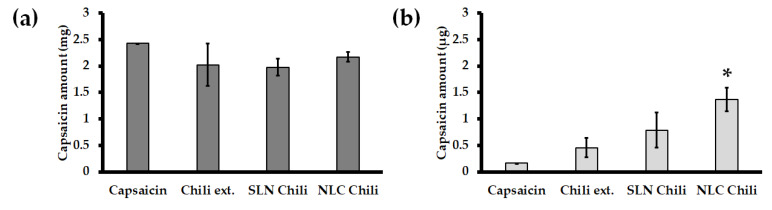
Distribution of capsaicin in the skin from capsaicin solution, chili extract solution, SLN chili and NLC chili. (**a**) The amount of capsaicin in the stratum corneum and (**b**) the amount of capsaicin in the dermis. Abbreviations: SLN, solid lipid nanoparticles; NLC, nanostructured lipid carriers. * The values are significantly different (*p* < 0.05) from other formulations.

Tape stripping and skin retention tests were conducted after an in vitro 24 h skin permeability study. Capsaicin concentration in the stratum corneum was determined in stillborn porcine skin by tape stripping to calculate the capsaicin concentration in the epidermis layer. The capsaicin concentration retained in the skin was determined by skin retention to reveal the capsaicin concentration in the dermis layer. The results from tape stripping showed that the capsaicin concentrations were non-statistically different (*p* > 0.05) in all formulations. However, the skin retention data showed that the capsaicin concentration from the chili extract-loaded nanoparticles significantly (*p* < 0.05) penetrated through the skin more than the chili extract and capsaicin solution, and particularly compared to the chili extract-loaded NLC. Skin retentions from chili extract-loaded SLN and NLC were approximately 1.75 times and 3 times greater than the chili extract, respectively. The amount of capsaicin from tape stripping and skin retention are presented in Figure 4a,b, respectively. The retention study could be described as the cumulative amount of capsaicin in the dermis layer, which is the target site of action for pain relief. The capsaicin concentration in the retention study showed that the chili extract containing a high dose of capsaicin (0.25%) was efficiently incorporated in the nanodelivery system, and particularly the NLC formulation. The enhanced skin retention of chili extract-loaded NLC provided the highest accumulation of capsaicin in the dermis layer more than SLN and the chili extract solution, due to its occlusive effect on the skin surface with high specific surface area and adhesive properties [10,16]. Moreover, the nanoparticle and cell membrane interaction contributed to chemical translocation and skin permeation enhancement, which depends on the nanoparticle properties, such as their material, size, shape, and surface charge [42,43].

### 3.5. Irritation Properties of Chili Extract Determined by the HET-CAM Assay

**Figure 5:**
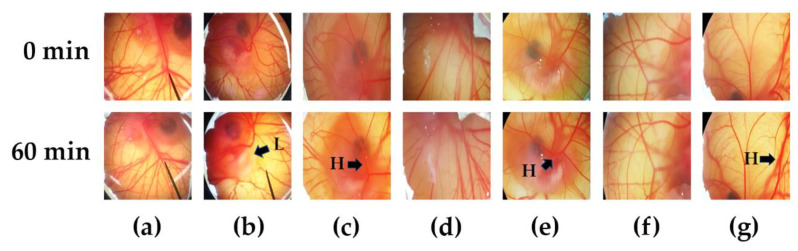
Photographs illustrating the effect of (**a**) the negative control (normal saline solution), (**b**) the positive control (1% SLS solution), (**c**) chili extract solution, (**d**) blank SLN, (**e**) chili extract-loaded SLN, (**f**) blank NLC, and (**g**) chili extract-loaded NLC in the hen’s egg test chorioallantoic membrane (HET-CAM) assay before applying the samples (upper panels) and at the end of the experiment (lower panels). Abbreviations: SLS, sodium lauryl sulphate; SLN, solid lipid nanoparticles; NLC, nanostructured lipid carriers; H, vascular hemorrhage; L, vascular lysis.

Additionally, safety is another concern of using nanodelivery systems. The HET-CAM assay is a predictive model for in vitro irritation by observing the vascular damage and calculating the irritation score (IS) [24]. This method has been widely used as an alternative method for skin irritation instead of an animal model since it is simple, rapid, sensitive, and inexpensive [23]. The CAMs were tested with normal saline solution, 1% SLS solution, and each of the samples are shown in Figure 5a–g, respectively. This method was well-validated since normal saline solution, which was used as the negative control, showed no irritation (IS = 0.00 ± 0.00), whereas 1% SLS solution, which was used as the positive control, showed severe irritation (IS = 10.98 ± 0.06). The blank formulations exhibited comparable IS scores to that of the negative control, while the IS scores of SLN_B and NLC_B were 0.73 ± 0.06 and 0.21 ± 0.04, respectively. The present study shows that the moderate irritation of chili extraction solution (IS = 6.77 ± 0.13) could be minimized to a mild level by encapsulation in SLN and NLC, with IS scores of 2.74 ± 0.04 and 2.48 ± 0.06, respectively. A likely explanation is due to the high entrapment efficiency and the controlled release behavior of SLN and NLC. Capsaicin was encapsulated within the solid matrix and gradually released from the nanoparticles, thus leading to less irritation in the HET-CAM assay. The results are in good accordance with previous studies that reported active compounds after encapsulation in SLN or NLC showed lower irritation than the solution form [44,45]. Therefore, SLN and NLC are potential nanocarrier systems for highly concentrated capsaicin incorporation. The systems were well-tolerated and safe for topical applications.

### 3.6. In Vitro Skin Irritation Test Using the EpiDerm^TM^ Model

**Figure 6:**
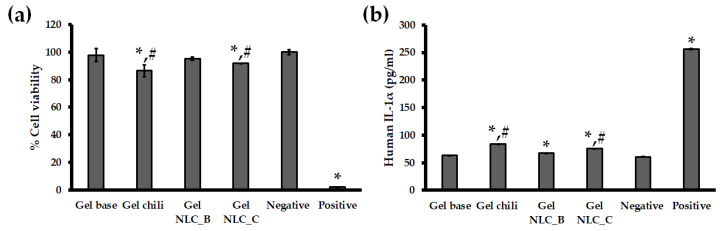
In vitro skin irritation test using EpiDerm^TM^ treated with gel based, gel chili, blank NLC in gel based formulation (Gel NLC_B), chili extract-loaded NLC incorporated in gel formulation (Gel NLC_C), negative control (Dulbecco’s phosphate buffered saline (DPBS)), and positive control (5% sodium dodecyl sulfate (SDS)). The EpiDerm^TM^ model was treated with samples and controls; (**a**) percentage (%) cell viability of EpiDerm^TM^, and (**b**) release of IL-1α from EpiDerm^TM^. Abbreviations: NLC, nanostructured lipid carriers; DPBS, Dulbecco’s phosphate buffered saline; SDS, sodium dodecyl sulfate. * The value is significantly different from the negative control (*p* < 0.05). # The value is significantly different between Gel NLC_C formulation and Gel Chili (*p* < 0.05).

With the endeavor of the scientists to procure validated irritation test protocols for irritancy prediction of wide variety of substances, the Draize skin irritation test in animals has been used for 80 years; however, some drawbacks such as the difference between rabbit and human responses have been reported [22]. Alternative to the Draize skin irritation test, the MTT (viability) and IL-1α measurement on the EpiDerm^TM^ reconstructed human epidermal (RHE) model are accepted as the in vitro skin experiments for the classification of irritation and corrosive chemicals based on the test guidelines of the Organization for Economic Cooperation and Development (OECD TG431 and TG439) [46,47]. The in vitro skin irritation test using the RHE model has been applied to test the skin irritancy of topical formulations such as gels, lotions, or creams. The commercial strength of 0.075% capsaicin was tested in this study. From the other experiments, as mentioned in Section 3.3, Section 3.4 and Section 3.5, the selected colloidal dispersion was NLC, so the gel formulation containing the chili extract-loaded NLC with 0.075% capsaicin was our sample of interest. The percentages of EpiDerm^TM^ cell viability after exposure to samples and controls were over 50%, indicating that all formulations were non-irritant [25,26], compared with 5% SDS (positive control), which showed strong irritancy. However, the percentage of cell viability from Gel NLC_C (91.85% ± 0.16%) was significantly (*p* < 0.05) higher than that of chili extract gel (86.49% ± 4.34%), revealing that the skin irritation of NLC was lower than the free form alternative, similar to the findings of previous reports [10,25]. The percentages (%) of cell viability are shown in Figure 6a. The concentrations of IL-1α, a proinflammatory cytokine, from the EpiDerm^TM^ after incubation with the samples and controls are presented in Figure 6b. The percentages of cell viability and IL-1α secretion from the incubation with the chili extract-loaded NLC and plain chili extract incorporated in gel formulations are significantly (*p* < 0.05) different from those of DPBS. The IL-1α from EpiDerm^TM^ treated with the Gel NLC_C and Gel NLC_B formulations was significantly increased (*p* < 0.05) compared with the negative control (DPBS), which is likely due to the effect of surfactant in the formulations [48].

## 4. Conclusions

Lipid-based nanocarriers with green chemistry production using the selection of a lipid core mixture and hot high-pressure homogenization for chili oleoresin entrapment were successfully developed. Optimized chili extract-loaded NLCs exhibited a spherical shape with a particle size of less than 200 nm and narrow size distribution. The benefits of natural chili extract are well-accepted in Ayurvedic remedies for pain. Attempts to entrap the major compound, capsaicin, from chili oleoresin were demonstrated in this study. The NLC formulation was more effective regarding encapsulation efficiency, in vitro capsaicin release, and permeability characteristics than the SLN formulation, enhancing the capsaicin delivery into the dermal layer of the skin. This optimized formulation was proven with the HET-CAM assay and EpiDerm^TM^ model, which showed that the chili extract-loaded NLC minimized the irritation compared to the chili extract formula. Therefore, the present study demonstrated that chili extract-loaded NLC is a promising delivery system for delivering high levels of capsaicin through the skin. Lower skin irritation of chili-encapsulated NLC highly depended on the significantly faster transdermal penetration of the dermis and prolonged capsaicin release from the permeated NLC. Nevertheless, further preclinical and clinical investigations for the study of irritation will confirm the effectiveness of chili extract-loaded NLC for minimizing skin irritation.

## Figures and Tables

**Table 1:** Particle size, polydispersity index (PDI) and surface charges of the solid lipid nanoparticle (SLN) and nanostructured lipid carrier (NLC) formulations prepared by high pressure homogenization method.

Formulations	Lipid Phase	Particle Size (nm)	PDI	Zeta Potential (mV)
SLN 1:4 *	GBH + COH	153.74 ± 6.72	0.39 ± 0.01	−44.26 ± 1.22
	GBH + COH + chili	176.80 ± 6.19	0.15 ± 0.07	−33.64 ± 1.09
	GMS + COH	129.42 ± 5.80	0.33 ± 0.05	−42.16 ± 0.77
	GMS + COH + chili	148.42 ± 4.80	0.22 ± 0.04	−40.78 ± 0.68
	GBH + SOH	154.68 ± 11.87	0.46 ± 0.01	−33.14 ± 1.31
	GBH + SOH + chili	154.66 ± 4.22	0.17 ± 0.02	−26.60 ± 1.84
	GMS + SOH	113.74 ± 3.58	0.43 ± 0.06	−34.94 ± 1.39
	GMS + SOH + chili	160.86 ± 5.47	0.19 ± 0.04	−28.24 ± 0.69
NLC 1 4 *	GBH + COH + IPM	150.98 ± 3.09	0.31 ± 0.02	−35.40 ± 1.10
	GBH + COH + IPM + chili	156.10 ± 2.82	0.11 ± 0.02	−31.40 ± 1.45
	GMS + COH + IPM	139.34 ± 3.95	0.38 ± 0.03	−34.64 ± 1.37
	GMS + COH + IPM + chili	148.50 ± 2.94	0.12 ± 0.03	−29.58 ± 1.37
	GBH + SOH + IPM	150.42 ± 4.12	0.43 ±0.03	−31.54 ± 1.96
	GBH + SOH + IPM + chili	154.86 ± 1.66	0.16 ± 0.05	−30.90 ± 0.94
	GMS + SOH + IPM	126.84 ± 4.61	0.38 ± 0.06	−30.92 ± 0.95
	GMS + SOH + IPM + chili	150.08 ± 2.16	0.16 ± 0.03	−27.10 ± 0.91

* The ratio of lipid mixture to surfactant in the formulations Abbreviations: GBH, glyceryl behenate; GMS, glyceryl monostearate; COH, cetyl alcohol; SOH, stearyl alcohol; IPM, isopropyl myristate.

**Table 2:** Particle size, polydispersity index (PDI), and zeta potential of lipid nanoparticles using different methods for particle size reduction with paired glyceryl monostearate (GMS) and cetyl alcohol (COH) as solid lipids.

Methods	Formulations	Particle Size (nm)	PDI	Zeta Potential (mV)
Probe sonication	SLN	1671.00 ± 643.07	0.97 ± 0.07	−45.18 ± 1.34
	NLC	502.24 ± 136.29	0.68 ± 0.08	−45.80 ± 0.83
High pressure homogenizer	SLN	129.42 ± 5.80	0.33 ± 0.05	−42.16 ± 0.77
	NLC	139.34 ± 3.95	0.38 ± 0.03	−34.64 ± 1.37

Abbreviations: SLN, solid lipid nanoparticles; NLC, nanostructured lipid carriers.

**Table 3:** Percentage of entrapment efficiency (EE) and loading capacity (LC) of chili extract-loaded SLN and NLC prepared by the high-pressure homogenization method.

Capsaicin Conc. (%)	Formulation	Lipid Phase (Wax)	% EE	% LC
0.075	SLN	GBH + COH + chili	78.33 ± 0.25	2.35 ± 0.01
		GMS + COH + chili	82.23 ± 1.69	2.47 ± 0.05
		GBH + SOH + chili	77.27 ± 5.98	2.32 ± 0.18
		GMS + SOH + chili	76.49 ± 8.85	2.29 ± 0.27
	NLC	GBH + COH + IPM + chili	85.99 ± 6.37	2.58 ± 0.19
		GMS + COH + IPM + chili	91.67 ± 4.02	2.75 ± 0.12
		GBH + SOH + IPM + chili	84.65 ± 3.01	2.54 ± 0.09
		GMS + SOH + IPM + chili	85.43 ± 1.29	2.56 ± 0.04
0.25	SLN	GMS + COH + chili	68.63 ± 0.54	6.86 ± 0.05
	NLC	GMS + COH + IPM + chili	85.27 ± 0.12	8.53 ± 0.01

Abbreviations: SLN, solid lipid nanoparticles; NLC, nanostructured lipid carriers; GBH, Glyceryl behenate; GMS, Glyceryl monostearate; COH, cetyl alcohol; SOH, stearyl alcohol; IPM, isopropyl myristate.

**Table 4:** The crystallinity index of solid lipid and freeze-dried formulations.

Samples	Onset (°C)	Peak (°C)	Endset (°C)	Integral (mJ)	ΔH (J/g)	% Crystallinity
GMS	57.81	66.77	70.83	−1043.38	−163.03	-
COH	48.06	51.35	55.69	−1291.74	−205.04	-
Physical mixture	49.61	52.15	56.11	−719.92	−110.76	100.00
SLN blank	34.75	37.88	40.97	−123.57	−14.54	65.64
NLC blank	34.88	38.72	42.81	−52.26	−6.15	39.66
SLN chili	30.91	39.84	45.24	−61.72	−7.09	15.33
NLC chili	30.49	35.21	44.19	−10.81	−1.26	3.13

Abbreviations: SLN, solid lipid nanoparticles; NLC, nanostructured lipid carriers; GMS, Glyceryl monostearate; COH, cetyl alcohol.

**Table 5:** Skin permeation parameters of capsaicin release from the chili extract and chili extract-loaded lipid-based nanoparticles. Data represent the mean ± SD.

Formulation	Flux (µg/cm^2^ h)	Lag Time (h)	Permeability Coefficient (cm/h) × 10^−5^	Q_24h_ (µg)	Enhancement Ratio (*E_r_*)
Chili extract	0.28 ± 0.05	5.95 ± 0.15	11.36 ± 1.90	9.05 ± 1.54	1.00 ± 0.00
SLN chili	0.37 ± 0.12	2.31 ± 0.07 *	17.17 ± 3.31 *	11.54 ± 1.78	1.39 ± 0.12
NLC chili	0.45 ± 0.16	2.30 ± 0.06 *	21.02 ± 4.38 *	13.68 ± 1.79 *	1.70 ± 0.17

Abbreviations: Q_24h_, cumulative capsaicin permeated through the skin at 24 h; SLN, solid lipid nanoparticles; NLC, nanostructured lipid carriers. * The value is significantly different (*p* < 0.05) than the chili extract (control).

## Supplementary Materials

The following are available online at https://www.mdpi.com/1999-4923/12/5/463/s1, Figure S1: Representative HPLC chromatograms of capsaicin (a) and chili extract (b), Table S1: The solidified inner crystal patterns of each solid lipid, Table S2: Regression coefficient (R2) values of the kinetic models.
