# Supplementary material for: Enhanced Transdermal Delivery of Concentrated Capsaicin from Chili Extract-Loaded Lipid Nanoparticles with Reduced Skin Irritation
Source: Pharmaceutics. 2020 May 19;12(5):463. doi: 10.3390/pharmaceutics12050463 (PMC7285225; doi:10.3390/pharmaceutics12050463)
Supplement: Supplementary file 1 [file pharmaceutics-12-00463-s001.pdf]

pharmaceutics

MDPI

Article

Enhanced Transdermal Delivery of Concentrated Capsaicin from Chili Extract-Loaded Lipid Nanoparticles with Reduced Skin Irritation

Phunsuk Anantaworasakul [1], Wantida Chaiyana [1,2], Bozena B. Michniak-Kohn [3], Wandee Rungseevijitprapa [4,*] and Chadarat Ampasavate [1,5,*]

Capsaicin contents in chili extract

The high-performance liquid chromatography (HPLC) chromatograms of capsaicin and dihydrocapsaicin show the retention times of 7.315 and 9.932 min, respectively.

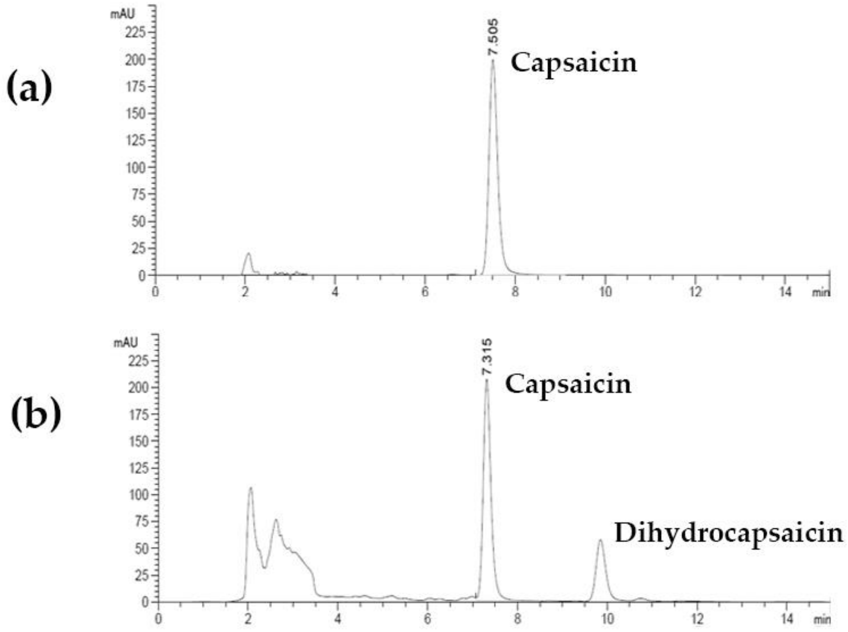

Figure S1. Representative high-performance liquid chromatography (HPLC) chromatograms of capsaicin (a) and chili extract (b).

Table S1. The solidified inner crystal patterns of each solid lipid.

Solid Lipid	m.p. (°C)	rHLB	5X	10X	20X
Glyceryl behenate	69-74	2	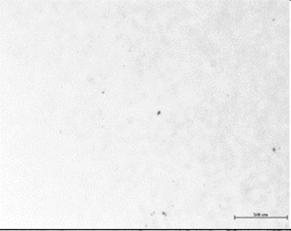	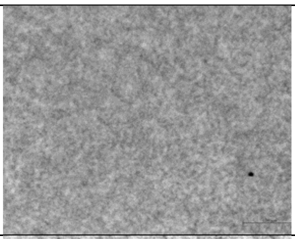	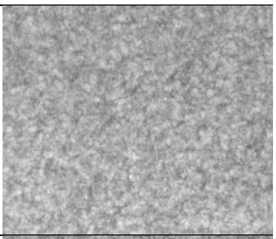
Glyceryl monostearate	55	3.8	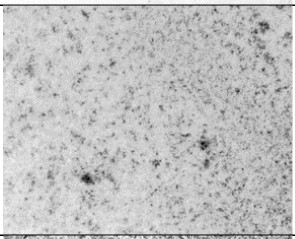	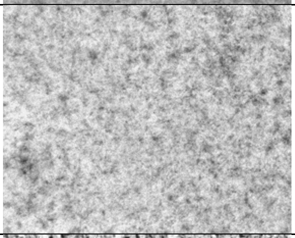	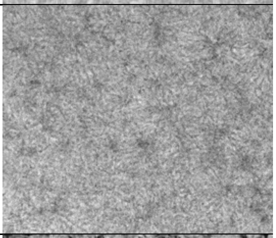
Stearyl alcohol	59.8	15.5	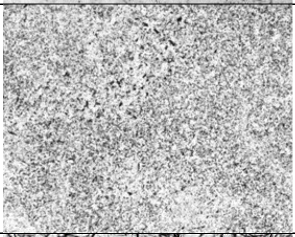	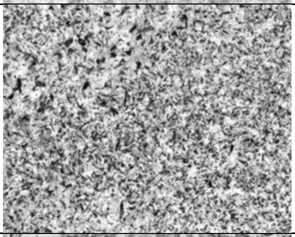	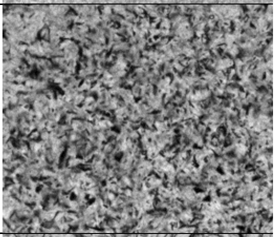
Cetyl alcohol	49.3	15.5	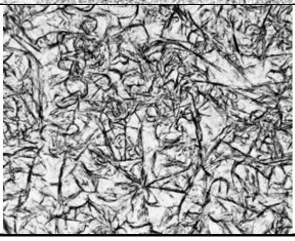	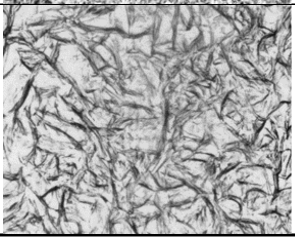	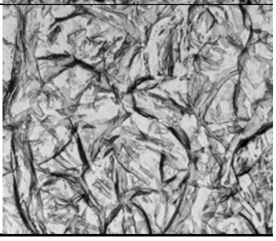

Table S2. Regression coefficient (R^2) values of the kinetic models.

Formulation	Zero-order	First-order	Higuchi
Capsaicin	0.9952	0.9201	0.9754
Chili extract	0.9918	0.8893	0.9786
SLN chili	0.9952	0.8459	0.9818
NLC chili	0.9941	0.8346	0.9841
